# An Activation Likelihood Estimation Meta-Analysis Study of Simple Motor Movements in Older and Young Adults

**DOI:** 10.3389/fnagi.2016.00238

**Published:** 2016-10-17

**Authors:** Ted K. Turesky, Peter E. Turkeltaub, Guinevere F. Eden

**Affiliations:** ^1^Center for the Study of Learning, Georgetown University Medical Center, WashingtonDC, USA; ^2^Interdisciplinary Program in Neuroscience, Georgetown University, WashingtonDC, USA; ^3^Neurology Department, Georgetown University Medical Center, WashingtonDC, USA; ^4^Research Division, MedStar National Rehabilitation Hospital, WashingtonDC, USA

**Keywords:** aging, finger, movement, motor, fMRI, PET, neuroimaging, meta-analysis

## Abstract

The functional neuroanatomy of finger movements has been characterized with neuroimaging in young adults. However, less is known about the aging motor system. Several studies have contrasted movement-related activity in older versus young adults, but there is inconsistency among their findings. To address this, we conducted an activation likelihood estimation (ALE) meta-analysis on within-group data from older adults and young adults performing regularly paced right-hand finger movement tasks in response to external stimuli. We hypothesized that older adults would show a greater likelihood of activation in right cortical motor areas (i.e., ipsilateral to the side of movement) compared to young adults. ALE maps were examined for conjunction and between-group differences. Older adults showed overlapping likelihoods of activation with young adults in left primary sensorimotor cortex (SM1), bilateral supplementary motor area, bilateral insula, left thalamus, and right anterior cerebellum. Their ALE map differed from that of the young adults in right SM1 (extending into dorsal premotor cortex), right supramarginal gyrus, medial premotor cortex, and right posterior cerebellum. The finding that older adults uniquely use ipsilateral regions for right-hand finger movements and show age-dependent modulations in regions recruited by both age groups provides a foundation by which to understand age-related motor decline and motor disorders.

## Introduction

Voluntary finger-movement tasks are commonly employed in neuroimaging studies. Thus far, the brain bases of this type of motor function have been well-characterized in young adults (Chouinard and Paus, 2006; Witt et al., 2008). For example, Witt et al. (2008) applied the activation likelihood estimation (ALE) meta-analysis method (Turkeltaub et al., 2002) to publications in which healthy young subjects performed a range of finger-tapping tasks. Of these, a subset of studies specifically used right-hand index finger-tapping tasks and were analyzed separately (Witt et al., 2008). This specific ALE map revealed likelihoods of activation in left primary sensorimotor cortex (SM1), supplementary motor area (SMA), ventral premotor cortex (PMv), basal ganglia, as well as bilateral anterior cerebellum, claustra, dorsal premotor cortex (PMd), and dorsolateral prefrontal cortex (DLPFC) and right inferior parietal lobule (IPL), insula, and inferior frontal gyrus (IFG). Importantly, these brain regions do not function in isolation, but are part of extensive efferent and afferent motor control pathways. One such pathway consists of upper motor neurons projecting from cortical motor areas to lower motor neurons in the spinal cord (via the cortico-spinal tract) to initiate movement. Afferent pathways originating at receptors in the skin, joints, and muscles feed back (e.g., about whether the target has been reached) to somatosensory cortex and cerebellum, which eventually project to cortical motor areas to improve subsequent movements.

While these investigations have provided a thorough understanding of simple finger-movement tasks in younger adults, motor processing in older adults is less well-understood. Filling this void is important, given that the motor system is affected by age (Seidler et al., 2010). Specifically, aging is associated with impairments in motor control, including gait, balance, and coordination; degeneration of neurotransmitter systems in putative motor regions (Seidler et al., 2010); and reductions in gray matter volume of motor system regions (Good et al., 2001; Hoffstaedter et al., 2015). A clear understanding of age-related differences in the motor systems of unimpaired populations can also provide a key baseline for contextualizing disease-related [e.g., in Parkinson’s disease (Yu et al., 2007) or amyotrophic lateral sclerosis (Poujois et al., 2013)] and neuroplastic [e.g., after stroke (Rehme et al., 2012)] changes to the motor systems. In addition, a reliable account of the pattern of activity underlying simple voluntary finger movements in older adults will aid in advancing conceptual models of age-related differences. Currently, age-related changes in activation in motor system brain regions are thought to be the consequence of one of two processes. One, described as compensation, posits that certain brain regions are upregulated in older adults to allow them to perform at a level commensurate with young adults. The second is de-differentiation, in which additional brain areas in older adults are recruited non-selectively, possibly as a consequence of age-related degeneration of inhibitory projections (for a review, see Seidler et al., 2010). Lastly, simple finger movements are a necessary part of commonly used paradigms in cognitive studies (in the form of a button-press response), with the baseline conditions not always controlling for the motor component (e.g., fixation). Thus, a difference in brain activity between young and older adults under these experimental conditions may be incorrectly attributed to cognitive function when it is the motor response that is the origin of the difference between the two groups.

Several studies directly comparing older and young adults have begun to address this gap by identifying brain areas underlying externally paced finger movements of the right hand (Calautti et al., 2001; Hutchinson et al., 2002; Mattay et al., 2002; Riecker et al., 2006; McGregor et al., 2009; Loibl et al., 2011). These studies required older [average ages between 59 (Mattay et al., 2002) and 71 (McGregor et al., 2009)] and young [average ages between 22 (McGregor et al., 2009) to 30 (Mattay et al., 2002)] adults to pace their movements in synchrony with an auditory or visual stimulus whose rate differed across studies [range: 0.6 Hz (Mattay et al., 2002) to 3.75 Hz (Riecker et al., 2006)]. Three (Mattay et al., 2002; Riecker et al., 2006; McGregor et al., 2009) of six of these studies reported that there were no differences in accuracy between older and young adults (however, older adults were relatively slower in all three studies). One study (Hutchinson et al., 2002) reported that performance (measured in terms of rate and degree of excursion) did not differ between the two age groups. One study (Loibl et al., 2011) reported that the groups were matched on performance, but didn’t specify the measure(s). The sixth study (Calautti et al., 2001) did not report on performance at all. These studies found that older compared with young adults showed more activity in several motor regions (**Table 1**), most notably in right cortical motor areas. For the reverse comparison of less activation in older compared with young adults, these same publications report a few regions: left SM1 (Hutchinson et al., 2002; Riecker et al., 2006), left LPMC (Hutchinson et al., 2002), left pre-SMA (Riecker et al., 2006), and bilateral cerebellum (Hutchinson et al., 2002). Taken together, while some brain areas (e.g., right SM1, bilateral or unilateral SMA/cingulate cortex, right LPMC) are reported to be more active in older compared with younger adults in most of the studies, results for other brain regions have been highly inconsistent, limiting the understanding of the effects of healthy aging on the functional neuroanatomy of the motor system. Further, when identifying age-related differences, it is important to tease apart which of these are due to a *relative* difference in activation between the two groups from those resulting from *unique* activity in one of the two groups (i.e., complete absence of activation in the other group). The interpretation of the above-described between-group differences can be aided by the within-group results reported from these same publications. Specifically, of these six aging studies, two showed unique recruitment of right SM1 in older adults (Mattay et al., 2002; Riecker et al., 2006), while two showed activation of this region in both older and young adults but relatively more for the older group (Hutchinson et al., 2002; Loibl et al., 2011). One study reported greater activation of right SM1 in older versus young adults but did not report within-group results (McGregor et al., 2009), making the distinction between unique and relative activation impossible to determine. Finally, one study did not report between-group differences in SM1 at all (Calautti et al., 2001). Taken together, whether aging is associated with unique, relatively greater (compared with young adults), or no engagement of ipsilateral SM1 remains to be answered.

**Table 1:** Locations of activations from fMRI and PET studies of the aging motor system.

Study	R. SM1	SMA^∗^	L. LPMC	R. LPMC	L. Cb	B. IPL	L. SPL	R. SPL	L. Put
Calautti et al., 2001				x					
Hutchinson et al., 2002	x	x					x		
Loibl et al., 2011	x	x	x	x	x		x	x	
Mattay et al., 2002	x	x	x	x	x	x			x
McGregor et al., 2009	x	x	x	x	x	x			
Riecker et al., 2006	x	x		x					

*R, right; L, left; B, bilateral; SM1, sensorimotor cortex; SMA, supplementary motor area; LPMC, lateral premotor cortex; Cb, cerebellum; IPL, inferior parietal lobule; SPL, superior parietal lobule; Put, putamen. ^∗^Activation in bilateral or unilateral SMA or cingulate cortex.*

The goal of the present study was to address these inconsistencies by providing a coherent picture of the aging motor system. We used the ALE meta-analysis technique (Turkeltaub et al., 2002) to identify robust concordance in activity underlying finger movement in young and older adults. The ALE method will find activations that are consistent across a range of studies utilizing different methods; however, when examining between-group differences, as we intend to do, experimental procedures need to be carefully matched so that the resultant between-group differences can be attributed to brain activation differences and not to one group containing a greater number of experiments using a particular experimental procedure (e.g., if our older group contained a substantially greater number of experiments that used auditory stimuli). Such careful study selection for ALE analyses inevitably results in a smaller number of eligible studies that is ultimately submitted for analyses (e.g., Engelmann et al., 2012). For example, only studies with right-hand finger movements generated in response to an externally paced stimulus were included in our analysis, as internally and externally paced movements engender differential brain responses and differential age-related effects (McGregor et al., 2009), and external pacing studies have the benefit of engendering similar levels of performance in older and young adults (Greene and Williams, 1993).

Indeed, many studies have used ALE to examine effects between populations, but have done so by using foci from between-group comparisons done in the original studies (for meta-analyses with comparable numbers of constituent studies, please see Amanzio et al., 2013; Ramage et al., 2013; Richlan et al., 2013 or, for VBM, Richlan et al., 2013), rather than by using within-group foci to generate separate ALE maps and then examining between-group effects. Here, we examined age-related differences in the motor system by performing between-group statistics on ALE maps generated from within-group data, rather than by generating ALE maps from coordinates of between-group effects. We first identified published papers containing experiments performed in older adults: four publications included studies that included groups of both younger and older adults, and four reported older adult data as controls for participants with disorders. We then selected from the relatively larger corpus of studies conducted in young adults to match the older adult studies that did not have a younger comparison group. Using these publications, we conducted a meta-analysis on the data reported for young adults and older adults, generating two separate (young and older adult) group maps. We then examined these maps’ commonalities as well as their differences to provide a quantitative review of the published literature on brain activity during finger movements for older compared with younger adults. This approach has two advantages over using between-group foci. First, within-group data is more common in the literature. And second, incorporating within-group data allows us to differentiate between brain areas with unique versus relative likelihoods of activation. However, given the relative novelty of this approach (Eickhoff et al., 2011), few studies have used this method (e.g., Engelmann et al., 2012).

## Materials and Methods

### Selection of Studies and Experiments

First, we searched PubMed^1^ and Google Scholar^2^ for articles with combinations of the following keywords: “fMRI,” “PET,” “neuroimaging,” “motor,” “finger,” “tapping,” “flexion,” “movement,” “hand,” “aging,” and “older.” We then selected only publications that met our criteria for inclusion in the ALE analyses (please see below). To ensure no other potential papers were overlooked, we searched these publications’ reference lists and reviewed publications that had cited these (via Google Scholar).

For the ALE analysis of both groups, young adults and older adults, activation foci were included if the publication met the following criteria: (1) subjects were healthy, right-handed adults; (2) the average ages of young adult cohorts were between 18 and 34 and/or the average ages of older adult cohorts were between 49 and 82 (while this range extends below the border between adulthood and older age indicated in neurocognitive aging (i.e., age 60; Reuter-Lorenz and Lustig, 2005), our range was chosen from the age ranges (or, when range was not reported, one standard deviation below/above the mean) of the individual subjects who participated in the four studies of the aging motor system (Calautti et al., 2001; Mattay et al., 2002; Riecker et al., 2006; Loibl et al., 2011)); (3) fMRI or PET was used; (4) results were reported in Talairach (Talairach and Tournoux, 1988) or Montreal Neurological Institute (MNI) stereotaxic space; and (5) activation foci were generated from whole-brain, rather than region-of-interest, analyses; (6) studies required subjects to perform some movement of the fingers (e.g., index finger flexion, multi-digit sequences) interspersed with a baseline (rest or fixation) period; (7) studies required subjects to respond to stimuli using their right hands only; and (8) studies employed a regularly paced external stimulus. To illustrate the restrictiveness of these criteria for the older adults, 42 studies remained after limiting the sample of studies to the first three inclusion criteria. We then removed 24 studies that did not report within-group coordinates, two studies that performed ROI analyses only, three studies with movements of non-finger or hand body parts (e.g., elbow or wrist), and four studies that employed self rather than external pacing. In addition, one reported coordinates only for a subset of the brain regions revealed by their between-group analysis. This left eight experiments with within-group data in older adults.

The number of studies fitting the above criteria was far greater for young than for older adults. Thus, we first found studies on older adults that fit the above criteria, and then selected studies on young adults to match on (1) stimulus frequency, (2) stimulus modality (i.e., auditory or visual), and (3) effectors used (e.g., right index finger or multi-digit sequence). All three of these aspects can modulate the amount of activation measured (Blinkenberg et al., 1996; Rao et al., 1996; Riecker et al., 2006; Witt et al., 2008), and the matching strategy minimized confounds that may otherwise have been introduced if the studies in older adults differed in this regard from the studies in younger adults. By the end of this procedure, our goal was to have two groups of studies, one for older and one for young adults, whose stimulus frequency, stimulus modality, and effectors used did not differ significantly between groups.

### Eligible Experiments

Our criteria for inclusion into our meta-analysis resulted in 16 experiments and 183 foci total: eight experiments with 86 foci in the young adult group (*n* = 94; mean age = 26.3 ± 3.66 years) and eight experiments with 97 foci in the older adult group (*n* = 93; mean age = 60.6 ± 5.69 years). Four of these experiments came from publications that directly addressed the aging motor system as described in the introduction (Calautti et al., 2001; Mattay et al., 2002; Riecker et al., 2006; Loibl et al., 2011), as these studies reported foci for the within-group analyses of the older adult group as well as the young adult group. Two other studies (Hutchinson et al., 2002; McGregor et al., 2009) were not included because they did not report the coordinates for within-group data. The four remaining older adult experiments were data from control groups reported in studies focused on disorders (Elsinger et al., 2003; Tombari et al., 2004; Sharma et al., 2009; Rehme et al., 2011). The four remaining young adult experiments (Kuhtz-Buschbeck et al., 2003; Agnew et al., 2004; Akhlaghi et al., 2012) came from the general motor literatures and were selected because their experimental conditions most closely matched the older adult studies already selected.

Based on these selection criteria, the older adult and young adult experiments were well-matched for their experimental parameters. Both the older adult and young adult experiments consisted of seven fMRI studies and one PET study. In all experiments, subjects used their right hands to perform either individual finger movements (two older and three young adult experiments), multi-digit movements (four older and four young adult experiments), or whole-hand closures (two older and one young adult experiment) in response to visual (two older and three young adult experiments) or auditory stimuli (six older and five young adult experiments). For these studies, subjects lay supine in the scanner and pressed buttons as instructed, following training of the task prior to the scan [one study (Mattay et al., 2002) did not report whether training was done]. Subjects viewed stimuli on the screen or were presented with auditory stimuli that served to indicate to them to make the motor movement. The rate at which the stimuli were presented (and hence at which subjects responded) varied across the studies. However, the overall average stimulus frequency was equated for between the older and younger adult studies [*t*(14) = 0.00246; *p* > 0.99], to ensure that these experimental parameters did not influence the ALE result [one of the studies’ stimulus frequencies included in this calculation is an average value because the experiment employed a range of frequencies in a task used to generate motor activity (Riecker et al., 2006)]. All subjects in all studies were right handed, except for in one study (Lehéricy et al., 2006), where one out of 12 subjects was left-handed (and as such there was one left-handed subject in the entire group of 93 subjects). Details of the original published experiments used in the meta-analysis are provided in **Table 2**.

**Table 2:** Meta-analysis dataset.

Studies	Imaging	*N*	Mean age	Stimulus frequency	Stimulus type	Foci	Effector	Contrast	Task description
**Young adults**					
Calautti et al., 2001	PET	7	24	1.26	Auditory	10	Finger	Tapping vs. Rest	Thumb-to-index
Mattay et al., 2002	fMRI	10	30	0.6	Visual	7	Finger	Finger vs. Rest	Four buttons arranged in diamond shape
Kuhtz-Buschbeck et al., 2003	fMRI	12	24	2	Auditory	4	Finger	Motor execution, Simple vs. Baseline	Thumb-to-finger multi-digit sequence
Agnew et al., 2004	fMRI	12	28	1	Visual	15	Finger	Movement vs. Rest	Thumb flexion
Lehéricy et al., 2006	fMRI	12	23	1	Auditory	11	Finger	Scale vs. Rest	Index flexion
Riecker et al., 2006	fMRI	10	23	3.75^∗^	Auditory	6	Finger	Main effects during index finger movement	Index tapping
Loibl et al., 2011	fMRI	18	25	1	Auditory	12	Hand	Main effects during 1 Hz fist clenching	Fist clenching
Akhlaghi et al., 2012	fMRI	13	33	0.66	Visual	21	Finger	Brain activity during single finger tapping	Thumb-to-(pre-specified) finger
**Older adults**					
Calautti et al., 2001	PET	7	60	1.26	Auditory	4	Finger	Tapping vs. Rest	Thumb-to-index
Mattay et al., 2002	fMRI	12	59	0.6	Visual	16	Finger	Finger vs. Rest	Four buttons arranged in diamond shape
Elsinger et al., 2003	fMRI	13	64	1.67	Auditory	5	Finger	Synchronized tapping vs. Rest, controls	Index tapping
Tombari et al., 2004	fMRI	10	49	1	Auditory	12	Finger	Move vs. Rest, controls	Multi-digit (II–V) simultaneous flex-extend
Riecker et al., 2006	fMRI	10	66	3.75^∗^	Auditory	8	Finger	Main effects during index finger movement	Index tapping
Sharma et al., 2009	fMRI	13	58	1	Auditory	14	Finger	Executed movement vs. Baseline, controls	Thumb-to-finger multi-digit sequence
Rehme et al., 2011	fMRI	11	63	1	Visual	16	Hand	Hand vs. Baseline, controls	Fist closures
Loibl et al., 2011	fMRI	17	67	1	Auditory	22	Hand	Main effects during 1 Hz fist clenching	Fist clenching

*^∗^Average of stimulus frequencies used.*

### ALE Methods

Three approaches were used: (1) group ALE maps of young and older adults separately, (2) a conjunction analysis identifying commonalities between these two ALE maps (Eickhoff et al., 2011), and (3) a contrast analysis identifying differences in ALE maps between groups (Eickhoff et al., 2011). We followed the procedures laid out in the user manual for GingerALE 2.3^3^ and briefly explained in a previous publication from our lab (Purcell et al., 2011). To ensure that multiple, tightly clustered foci from a single experiment did not bias the MA maps, we applied the Non-Additive method in which, for each voxel in the brain, only the probability associated with the focus with the shortest Euclidean distance from that particular voxel (i.e., the maximum probability) was used for the MA (Turkeltaub et al., 2012). Significance of the output ALE map was assessed to distinguish true convergence across studies from random convergence (i.e., noise) and was undertaken using the cluster-level inference method, recommended for optimal sensitivity and as an alternative to the more stringent family-wise error correction (Eickhoff et al., 2012). The cluster-level inference threshold was set to *p* < 0.05 with a cluster-forming threshold false discovery rate (FDR; Thomas Nichols) ^4^ of pID < 0.05, assuming independence or positive dependence, and 10,000 permutations. GingerALE was used to identify peak MNI coordinates associated with all clusters in young and older adult maps, transform these coordinates into Talairach space using icbm2tal (Lancaster), and label nearest gray matter using the Talarach Daemon^5^.

To identify brain areas likely to be activated in both groups, and brain areas that differed between the two groups, we generated a conjunction map as well as two contrast ALE maps (older adult > young adult and young adult > older adult; Eickhoff et al., 2011). The conjunction map showed voxels that survived correction in both the individual group maps for young and older adults with a minimum cluster size of 100 mm^3^. Contrast maps (older adult > young adult and vice versa) were generated by directly subtracting one group ALE map from the other. Specifically, GingerALE was used to perform the following steps: (1) randomly re-group the experiments constituting the group datasets for young and older adults into two new datasets of the same size; (2) create ALE images for each of the new datasets and subtract one from the other; (3) repeat this process 10,000 times (number of permutations) to generate an extensive null distribution; and (4) test the actual ALE subtraction against this resultant null distribution using a voxel-wise FDR correction (Laird et al., 2005) of pID < 0.05 and a minimum cluster size of 100 mm^3^.

Results were visualized using the Mango software package^6^ with the Colin brain template in MNI space (Holmes et al., 2014). Functional motor regions (e.g., SM1, SMA, etc.) were labeled using the Human Motor Area Template (HMAT) depicted in axial slices (Mayka et al., 2006). This template was derived by implementing the ALE method on 126 fMRI or PET studies involving motor control, and demarcates three main divisions of motor areas [SM1, medial premotor cortex (MPMC), and LPMC] as well as their subdivisions [primary motor cortex (M1) and primary somatosensory cortex (S1) for SM1, SMA and pre-SMA for MPMC, and PMv and PMd for LPMC]. For our reporting, we differentiated the subdivisions of MPMC and LPMC, but not SM1, because most of the studies used in this meta-analysis showed activation in M1 and S1, and those activations registered in a single cluster. This is different from activations in MPMC and LPMC, which often registered in one but not both subdivisions. The areas demarcated by the template are inherently probabilistic in nature due to the ALE algorithm used to identify them. Probability maps for main and subdivisions represent the likelihood that an activation focus falls within a given area. We reported functional regions based on a 95% probability; i.e., if a coordinate fell within the 95% bounds for only one main or subdivision, we labeled that coordinate as that main or subdivision. Boundary zones (i.e., regions of the map in which main or subdivisions overlapped) manifest U-shaped probabilities, where the nadir represents an equal probability that an activation focus lies in adjacent main or subdivisions. We labeled coordinates in these boundary zones as the main or subdivision with the higher probability. Any coordinates that fell almost perfectly at the nadir were labeled with both names (e.g., pre-SMA/SMA).

## Results

### Within-Group ALE Analyses

A full list of ALE peaks (in MNI stereotaxic space) for the young and older adult groups is reported in **Table 3**, along with anatomical and functional labels. Superscript markings appending certain functional motor brain regions indicate that the peak is in a boundary zone between two main divisions (%1), e.g., between SM1 and LPMC; or subdivisions (%2), e.g., between PMv and PMd, as demarcated by the HMAT (see Materials and Methods).

**Table 3:** Activation likelihood estimation (ALE) peaks for within-group contrasts for right hand movements.

Anatomical region	Functional motor region	BA	Peak coordinates			*k*	Z (x 10^-3^)
			*x*	*y*	*z*		
**Young adults**					
Left post-central gyrus	SM1	3	-38	-22	52	9208	26.7
Right medial frontal gyrus	Pre-SMA/SMA	6	6	8	64	6536	11.6
Left inferior parietal lobule	40	-52	-38	30	712	6.89
Left insula		13	-48	-24	18	1112	10.2
Right insula		13	54	-22	18	1480	8.95
Left putamen			-22	6	8	3232	15.0
Right anterior cerebellum			18	-52	-22	6176	14.7
Left anterior cerebellum			-36	-58	-28	1720	10.2
Left thalamus			-14	-18	2	3744	18.5
							
**Older adults**					
Left post-central gyrus	SM1	2	-44	-22	52	11008	17.2
Right precentral gyrus	SM1^%1^	6	40	-10	60	4552	13.4
Left medial frontal gyrus	SMA	6	-4	-4	54	5672	13.6
Right inferior frontal gyrus	PMv	44	62	10	21	1552	12.6
Left inferior frontal gyrus	PMv	44	-54	2	18	720	7.90
Right inferior parietal lobule		40	40	-32	36	2880	13.1
Left superior parietal lobule		7	-34	-44	56	1000	10.2
Right insula		13	54	-18	18	888	8.13
Left inferior occipital gyrus		18	-30	-90	-4	888	7.77
Left putamen			-24	-8	6	848	7.31
Right putamen			24	4	-4	1016	11.2
Right anterior cerebellum			18	-52	-20	3544	22.9
Left anterior cerebellum			-26	-54	-22	1648	13.6
Left thalamus			-16	-18	0	1488	7.72

*^%1^ % at first level.*

#### Young Adults

**FIGURE 1:**
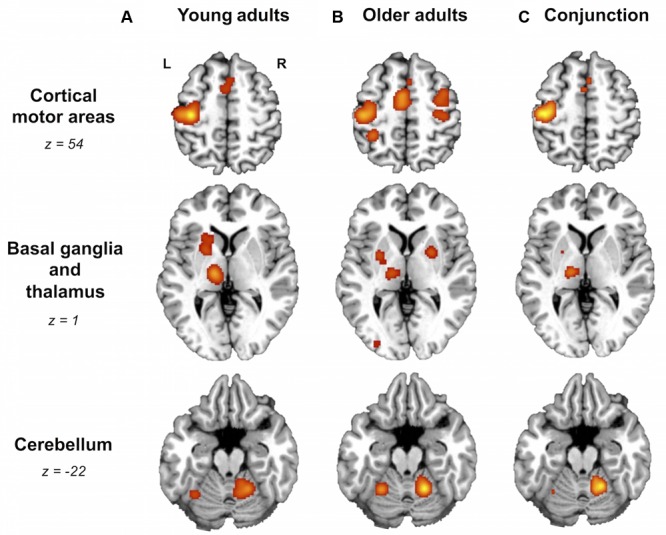
**Older and young adults likely recruit different brain areas.** Whole-brain activation likelihood estimation (ALE) maps for **(A)** young adults, **(B)** older adults, and **(C)** conjunction of older and young adults (cluster-level inference corrected threshold at *p* < 0.05). All maps exhibited suprathreshold ALE values in left primary sensorimotor cortex (SM1), left supplementary motor area (SMA), bilateral insula, right anterior cerebellum, and left thalamus. In addition, older and young adult maps showed suprathreshold ALE values in regions of right SMA, dorsal premotor cortex, and left putamen. In the older adult map only, please note the suprathreshold ALE values in right SM1, extending into dorsal premotor cortex. L, left hemisphere; R, right hemisphere. **Tables 3** and **4** provide the full list of ALE peaks for these maps.

For right-hand finger movement in young adults, we observed significant likelihoods of activation in left SM1 extending into PMd^%1^, bilateral pre-SMA, left SMA, right SMA, left IPL, bilateral insula, left putamen, left thalamus, and bilateral anterior cerebellum (**Figure 1A**).

#### Older Adults

For right-hand finger movement in older adults, the ALE map showed likelihoods of activation in left SM1 extending ventrally into left insula, right SM1^%1^ extending into PMd^%1^, left SMA extending into right SMA as well as left and right pre-SMA, bilateral PMv, right IPL, left superior parietal lobule, right insula, left inferior occipital gyrus, bilateral putamen, left thalamus, and bilateral anterior cerebellum (**Figure 1B**).

### Conjunction ALE Analysis

Areas most likely activated by both young and older adults, as revealed by conjunction analysis, included left SM1 extending into left PMd^%1^, left SMA extending into right SMA^%2^ and bilateral pre-SMA^%2^, bilateral insula, left thalamus, and right anterior cerebellum (**Figure 1C**; **Table 4**).

**Table 4:** Activation likelihood estimation peaks from conjunction of young and older adult ALE maps.

Anatomical region	Functional motor region	BA	Peak coordinates			*k*	Z (x 10^-3^)
			*x*	*y*	*z*		
Left post-central gyrus	SM1	3	-42	-22	52	5872	16.5
Left medial frontal gyrus	SMA	6	-6	-6	58	944	7.42
Left insula		13	-48	-26	20	936	9.08
Right insula		13	54	-20	18	232	7.16
Right anterior cerebellum			18	-52	-22	2592	14.7
Left thalamus			-16	-18	0	1224	7.72

### Between-Group ALE Analyses

**FIGURE 2:**
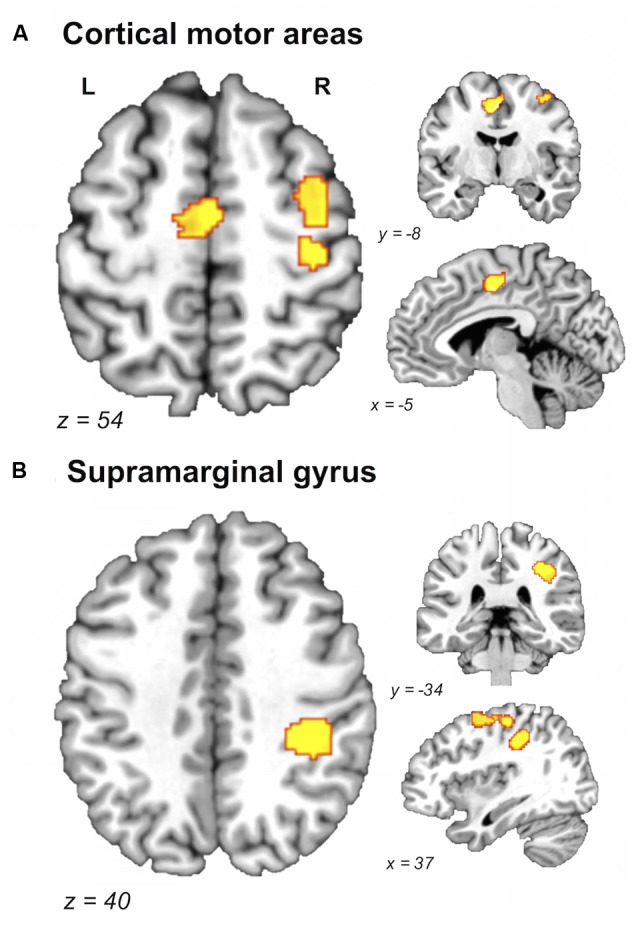
**Brain areas showing greater likelihood of activation in older compared with young adults.** Whole-brain ALE maps for the older > young adult contrast (voxel-wise FDR correction at *p* < 0.05, *k* > 100 mm^3^). Suprathreshold ALE values were observed in right primary sensorimotor cortex, right dorsal premotor cortex, and supplementary motor area **(A)**, and right supramarginal gyrus **(B)**. L, left hemisphere; R, right hemisphere. **Table 5** provides the full list of ALE peaks for this map.

**FIGURE 3:**
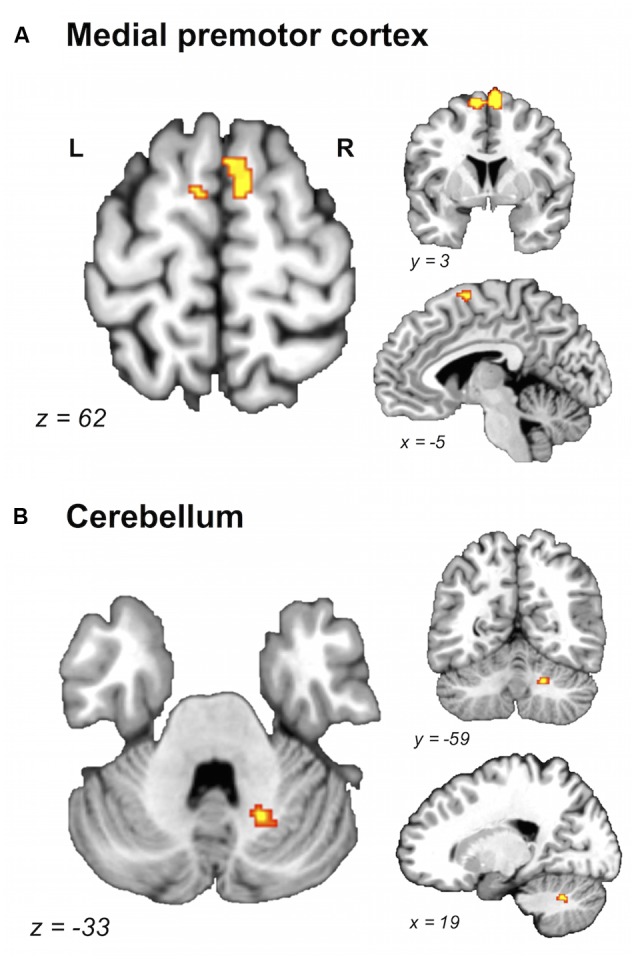
**Brain areas showing greater likelihood of activation in young compared with older adults.** Whole-brain ALE maps for the young > older adult contrast (voxel-wise FDR correction at *p* < 0.05, *k* > 100 mm^3^). Suprathreshold ALE values were observed in antero-dorsal regions of medial premotor cortex **(A)** and right cerebellum **(B)**. L, left hemisphere; R, right hemisphere. **Table 5** provides the full list of ALE peaks for this map.

A direct between-group comparison revealed that older compared with young adults had a greater likelihood of activation in right SM1 extending into PMd^%1^, right supramarginal gyrus (SMG), and left postero-ventral SMA extending into right SMA (**Figure 2**; **Table 5**). The reverse comparison revealed significantly lower ALE values in older compared with young adults in right pre-SMA, extending into left pre-SMA^%2^, and left SMA. Older adults also had significantly lower ALE values in right posterior cerebellum (**Figure 3**).

**Table 5:** Activation likelihood estimation peaks for between-group contrasts for right hand movements.

Anatomical region	Functional motor region	BA	Peak coordinates			*k*	Z (x 10^-3^)
			x	y	z		
**Young Adults > Older Adults**					
Right superior frontal gyrus	pre-SMA	6	6	14	68	1984	2.30
Right posterior cerebellum			18	-58	-34	136	2.09
							
**Older Adults > Young Adults**					
Right post-central gyrus	SM1	3	40	-21	54	2200	2.39
Left cingulate gyrus	SMA	24	-2	-6	47	1976	2.77
Right supramarginal gyrus^∗^		40	44	-38	32	2352	2.24

*^∗^Extreme was outside gray matter. This is the secondary extreme.*

## Discussion

This is the first quantitative meta-analysis of functional neuroimaging data reported for right-hand finger movements in older adults, and the first investigation into the commonalities and differences between activation likelihood maps generated for older and young adults. Our ALE analysis drew on studies in which subjects performed right hand index finger movements, multi-digit movements, or whole-hand closures, with the type of task equally distributed over the young and older adult groups. While there are task-dependent differences in brain activity for these paradigms (Witt et al., 2008), our focus was on age-dependent difference; thus, task type was balanced for the conjunction and between-group analysis. The results, based on eight experiments reported in older adults and eight experiments reported in young adults, revealed that both (via conjunction) groups engage left SM1 (extending into PMd), left SMA (extending into right SMA and bilateral pre-SMA), bilateral insula, left thalamus, and right anterior cerebellum. However, other aspects of the analyses revealed that older adults use additional brain regions that are not engaged by younger adults: a likelihood of activation was observed ipsilateral to the side of movement in right SM1 (extending into PMd) and SMG in the older adults within-group analysis, as well as in the between-groups contrast of older versus young adults. This observation—that older adults showed likelihood of activation in right SM1 and SMG when young adults did not—indicates unique recruitment of an ipsilateral network with older age. Another age-related finding was in the SMA, but here the results indicate the possibility of an age-dependent anatomical shift in brain activation. Specifically, both groups individually showed concordance across studies in the SMA; however, the particular subregion recruited differed depending on the age group, with young adults tending to use pre-SMA and older adults likely to activate more postero-ventral SMA. Finally, older compared to young adults had relatively less concordance in right posterior cerebellum.

### Young Adults and Older Adults Within-Group Findings

Our ALE results for Movement > Rest contrasts in young adults were similar to those from a prior meta-analysis of 23 published studies restricted to right-hand index finger movements [one of several meta-analyses reported by Witt et al. (2008), with the main meta-analysis of 38 studies including left hand movements]. They too observed left SM1, left PMd, bilateral SMA, right insula, left basal ganglia, and bilateral anterior cerebellum. It should be noted that our ALE map additionally identified left IPL, left insula, and left thalamus, whereas we did not identify some areas reported by Witt et al. (2008), namely right PMd, left PMv, right IPL, bilateral claustra, right IFG, and bilateral DLPFC. There are three notable methodological differences between the meta-analysis presented here and the comparable analysis presented by Witt et al. (2008). The first is the number of experiments entered into the analyses (eight for ours and 23 for Witt et al., 2008). Second, our meta-analysis included some experiments in which subjects performed finger movements involving multiple fingers, whereas the subset meta-analysis (23 studies) reported by Witt et al. (2008) only included index finger movements. And third, our ALE maps were generated from studies that used only externally paced stimuli (i.e., externally guided by auditory or visual stimuli), which, based on their predominant representation in Witt et al. (2008), seems to be the more common approach found in the literature. In contrast, Witt et al. (2008) generated their ALE map for right-hand index finger movements from 23 studies comprising experiments using *both* externally paced (23 experiments) and internally paced (self-guided with no external stimuli, 11 experiments) finger movements, while in another aspect of their study showing that likelihoods of activation seem to differ depending upon whether the movement is externally or internally driven.

Turning to the older adults, we found likelihoods of activation in this group bilaterally in a number of regions including SM1, LPMC, SMA and pre-SMA, posterior parietal cortex, putamen, and anterior cerebellum. These findings are consistent with two studies of older adults not included in our meta-analysis (because the coordinates of activation foci were not reported), which reported activation in similar ipsilateral, right cortical motor areas (Hutchinson et al., 2002; McGregor et al., 2009).

### Similarities and Differences between Young and Older Adults

A conjunction analysis revealed that older and young adults are both likely to recruit left SM1, left SMA, bilateral insula, left thalamus, and right anterior cerebellum. However, the between-group comparison revealed that older adults show a greater likelihood of engaging right SM1 (and extending into right PMd) and SMG, as well as bilateral postero-ventral SMA. When considered in the context of the results from the young adult within-group map, it becomes evident which of these regions are uniquely recruited by older adults and which regions are also present in young adults but demonstrate a relatively greater concordance in older compared to young adults. Interestingly, the right SM1 and right SMG, observed in both the older adults within-group map and the older versus young adults contrast, were not observed in the young adult map, indicating that these regions are uniquely recruited in older adults.

#### Primary Sensorimotor Cortex

SM1 comprises primary motor cortex (M1), the area considered to execute voluntary movement through its direct connection to the spinal cord (via the cortico-spinal tract), and primary somatosensory cortex (S1), the brain’s main area for receiving information about touch. Reliability of our findings in this area can be traced back to output from GingerALE, which provides information on the number and identity of the input foci that contribute to significant ALE clusters. All eight experiments contributed to the older adults’ within-group convergence in right SM1 (19 foci total). As young adults did not show a likelihood of activation in this area, no input foci from young adult data contributed to convergence in this area for the young adult within-group map. Consistent with this finding, two out of the four aging studies (Mattay et al., 2002; Riecker et al., 2006) whose within-group data were used in our meta-analysis, as well as one study not included in our ALE analysis (McGregor et al., 2009), also showed that older adults uniquely activate right SM1. In addition, one out of the four aging studies included in this meta-analysis (Loibl et al., 2011) and one not included (Hutchinson et al., 2002) showed greater activation in SM1 in older compared with young adults contrasts. These findings are also in line with a report that activation measured with fMRI in right M1 during a right-hand finger-tapping task is positively correlated with age (Naccarato et al., 2006).

Looking at a different literature, studies of age-related changes in connectivity parallel these observations of age-dependent recruitment of right SM1 ipsilateral to the side of movement. In a diffusion tensor imaging (DTI) study, functional anisotropy (FA), a proxy for white matter microstructure, was shown to change over the lifespan following an inverted U-curve in the body of the corpus callosum (Lebel et al., 2012). This tract includes fibers connecting the motor cortices of the left and right hemisphere (as well as other, non-motor regions). A DTI study exclusively examining the fibers of the body of the corpus callosum connecting homologous motor cortices found reduced FA in older compared with young adults (Fling and Seidler, 2012; Fling et al., 2012). These connections contribute to the inhibition of ipsilateral M1 by contralateral M1 during movements (i.e., transcallosal inhibition; Ferbert et al., 1992; Meyer et al., 1995, 1998; Netz et al., 1995; Boroojerdi et al., 1996; Gerloff et al., 1998; Stinear et al., 2001; Daskalakis et al., 2002; Duque et al., 2007). Specifically, it is believed that glutamatergic transcallosal fibers mediate local GABAergic circuitry (i.e., intracortical inhibition) that suppresses corticospinal neurons in ipsilateral M1 (for a review, see Conti and Manzoni, 1994). It is this mechanism that is thought to give rise to BOLD signal decreases in ipsilateral SM1 that have been observed during movement execution (Ferbert et al., 1992; Allison et al., 2000; Liepert et al., 2001; Hamzei et al., 2002; Newton et al., 2005). Relevant to the focus of this study, it follows that as transcallosal inhibition (Talelli et al., 2008) and intracortical inhibition (Peinemann et al., 2001) decrease from young to older adulthood, ipsilateral SM1 would show attenuation of the BOLD signal decrease or perhaps even a positive BOLD signal in older adults. This is consistent with our observation of a unique likelihood of activation of right SM1 in older adults.

It is worth considering whether differences in gray matter volume (GMV) may also underlie the effects we observed in right SM1. This explanation seems highly unlikely since gray matter atrophy, particularly reduced GMV in SM1, occurs with age (Good et al., 2001; Hoffstaedter et al., 2015), and therefore one might expect partial volume effects to contribute to fMRI signal loss (rather than gain) in the older adult group. In other words, such differences in brain structure would only explain less likelihood of activation in the older versus young adult comparisons. Since we found older adults to be more likely to activate in right SM1 and SMG, these age-related differences cannot be explained in terms of gray matter loss. As such, it seems that our observation of unique likelihood of SM1 activity in older adults occurs *despite* this region having experienced atrophy. It is also telling that we did not find differences between the groups in left SM1, even though left SM1 also contains less gray matter in the older adults (Good et al., 2001; Hoffstaedter et al., 2015). Altogether, it appears that gray matter decline in SM1 and SMG specifically cannot explain our finding, though it is possible that age-related gray matter atrophy in other brain regions contributes to functional reorganization in these areas.

#### Supramarginal Gyrus

Through its fronto-parietal circuitry, SMG is thought to contribute to integrating sensory information to subsequently guide motor output (Geyer et al., 2000). Five foci from four experiments (Mattay et al., 2002; Tombari et al., 2004; Sharma et al., 2009; Loibl et al., 2011) drove convergence in right SMG in our older adults within-group analysis. One of the aging studies used in our analysis showed greater recruitment of this area in an older compared with young adults contrast (Mattay et al., 2002), although unlike our results, their findings demonstrated that right SMG was not uniquely activated by older adults, but rather relatively more so compared with young adults. An aging study not included in this meta-analysis also showed relatively greater activations in IPL in older compared with young adults (McGregor et al., 2009), but whether the brain area was localized to SMG or angular gyrus specifically remains unclear. Interestingly, the foci that contributed to the SMG ALE cluster are from studies that required subjects to move multiple digits, as opposed to single digits. This may suggest that right SMG is uniquely recruited by older adults on multi-digit tasks or, as our meta-analysis had one more multi-digit experiment in the set of older adult studies, that the convergence in right SMG is an artifact of having one more multi-digit study in the older adult group. However, as two out of the five foci that contributed to the SMG cluster were from those studies in which older and young adults performed the same task, we think that the unique convergence in right SMG reflects true differences in older and young adults performing multi-digit tasks (and not an effect of having one more multi-digit experiment in the older group). We did not notice that task type or stimulus modality related to any other ALE findings. We also note the convergence of both SMG and PMd, the latter of which emerged from the contributions of two foci from a right-hand index finger, rather than multi-digit, task (Riecker et al., 2006). The appearance of these areas for either task suggests that older adults might uniquely rely on right, ipsilateral parieto-frontal areas to complete right-hand finger movements in general. Importantly, these areas are consistent with the well-characterized parieto-frontal circuitry revealed in the macaque, which projects from parietal cortex, including IPL, to LPMC and on to M1, and is thought to underlie the transformation of sensory input into motor output (Geyer et al., 2000). In contrast to many studies of the aging motor system, which, as described earlier, demonstrate unique activation in ipsilateral SM1 in older compared with young adults, this finding suggests that older adults may rely on a larger ipsilateral network, consisting not only of SM1, but also PMd and SMG. As we only included studies employing the right hand, it remains unclear whether this age-related pattern is unique to right hand movements, and if it is not, whether it is left- or right-lateralized. A meta-analysis similar to the one we conducted, but using data from studies employing left hand tasks, is needed to answer this question.

#### Supplementary Motor Area

**FIGURE 4:**
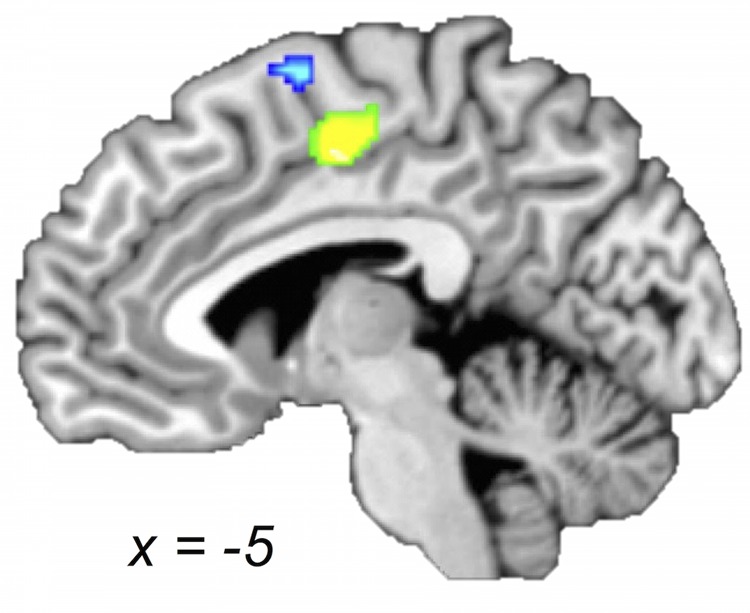
**Older and young adults likely recruit different regions of medial premotor cortex.** Midsagittal section (*x* = -5) of older > young adult (green) and young > older adult (blue) ALE contrasts (voxel-wise FDR corrected at *p* < 0.05). Young compared with older adults showed a greater likelihood of activation in a cluster extending into pre-SMA, whereas older compared with young adults showed a greater likelihood of activation in a postero-ventral region of SMA.

Supplementary motor area has been linked to a variety of motor behaviors including motor planning, sequence performance, and bimanual coordination (for a review, please see Nachev et al., 2008). Considering the SMA concordances, we found an interesting age-dependent pattern: both within-group and both between-groups maps revealed likelihoods of activation within SMA; however, with the exception of a small portion (as shown in the conjunction), the part of SMA identified in the young adults within-group map and in the young-greater-than-older-adults between-group map was spatially distinct from the region of SMA found in the group map of older adults and the older-greater-than-young-adults comparison (**Figure 4**). Specifically, bilateral postero-ventral SMA was more strongly recruited by the older adults and pre-SMA was more strongly recruited by the young adults. Three of the four aging studies (i.e., comparing young and older adults) used in this meta-analysis reported between-group differences in bilateral SMA, with most showing greater activation in older compared with younger adults (Mattay et al., 2002; Riecker et al., 2006; Loibl et al., 2011), while one showed the opposite (Riecker et al., 2006). In addition, two aging studies not included in our meta-analysis also showed greater activation in older adults in this area (Hutchinson et al., 2002; McGregor et al., 2009). Our observations are best described as a shift in concordance from pre-SMA to postero-ventral SMA with increasing age. The SMA has often been discussed in the context of task complexity (Chouinard and Paus, 2010). It has also been proposed that an anterior–posterior continuum exists along these medial premotor areas, with pre-SMA (more anterior) areas more involved in higher-order tasks and SMA proper more involved in relatively lower-order tasks (Picard and Strick, 1996; Nachev et al., 2008). As such, the question arises whether the observations in SMA can be attributed to task complexity. Since we were careful to control for task complexity in the selection of the studies, it seems that the older adults were recruiting postero-ventral SMA (associated with lower-level task processing) to complete the motor movements included in this meta-analysis. This may seem rather surprising, as one might expect these tasks to be harder for older adults. Perhaps the recruitment of pre-SMA becomes prohibited with age, or unnecessary due to other compensatory changes in the aging brain in support of motor tasks. It is also important to note that different anatomical connectivity profiles also distinguish these areas, with pre-SMA relatively more connected with frontal cortex and SMA relatively more connected with parietal cortex (Nachev et al., 2008). Thus, the co-occurrence of this posterior shift with the convergence in inferior parietal cortex (i.e., the SMG finding) for older compared with young adults may be indicative of a switch from top–down to bottom–up processing.

A closer look at the relationship between task performance and brain activity in the aging studies used in our meta-analysis is relevant in the context of this discussion. Two of the four studies reported significantly slower reaction times for the older compared with young adults (Mattay et al., 2002; Riecker et al., 2006), and in one of these studies this observation was disregarded because reaction times were only slower at the beginnings of runs (Riecker et al., 2006). Both studies reported no differences between the groups on accuracy. A third study reported no significant differences on reaction time or accuracy (Loibl et al., 2011), and the fourth did not report performance at all (Calautti et al., 2001). The other four studies used in our meta-analysis to generate the older adult ALE group map cannot be considered for this matter because they were not accompanied by a young adult comparison group (see Selection of Studies and Experiments; Eligible Experiments). Overall, it seems that in-scanner performance was only slightly different between groups, if at all, which makes it reasonable to conclude that task performance is not likely to explain the ALE map differences we observed. This is consistent with behavioral literature, which has shown no significant age-related differences in finger tapping (Greene and Williams, 1993) but pronounced age-related differences on more demanding tasks (Ruff and Parker, 1993; Smith et al., 1999). This underscores our rationale for including only studies that employed tasks of similar difficulty.

#### Cerebellum

The cerebellum detects differences between actual and intended movements. Through its various projections to upper motor neurons in the cerebral cortex and brainstem, it subsequently corrects for these differences (Purves et al., 2004). Our results show that the right posterior cerebellum exhibited relatively less concordance for older compared with younger adults. Although none of the studies used in our meta-analysis found age-related differences in this region, Hutchinson et al. (2002) (not included in our meta-analysis) also found less activation in cerebellum in older compared with young adults, in their case bilaterally (Hutchinson et al., 2002). Other than this, however, our finding is somewhat inconsistent with previous aging studies. Two studies included in this meta-analysis (Mattay et al., 2002; Loibl et al., 2011) reported unique activation in the older compared with young adults in left cerebellum, as did one study not included in the ALE analysis (McGregor et al., 2009). We are not certain what contributed to the discrepancy between our result and the results of other aging studies, but we note that age-related reductions in bilateral cerebellar GMV and WMV (e.g., Koppelmans et al., 2015) could support the differences we find. Notably, our finding was localized to more posterior aspects of the cerebellum or on the cusp of posterior and anterior cerebellum. Unlike anterior cerebellum, which is considered to be involved in motor control, the posterior cerebellum is pertinent for cognitive tasks (Allen et al., 1997; Schmahmann and Sherman, 1998). As such, it is tempting to speculate, akin to recruitment of posterior SMA and SMG, that older adults, compared with younger adults, do not have the option or the need to draw on regions that may reflect more complex processing.

### Relevance to Current Models of the Aging Motor System

Two main theories have been proposed to explain the additional activations observed in older compared with young adults. The first is compensation, in which better task performance corresponds to activation in additional brain areas (Mattay et al., 2002; Heuninckx et al., 2008). Indeed, Mattay et al. (2002) reported positive correlations between performance and bilateral SM1, LPMC, SMA, and left parietal cortex and cerebellum (Mattay et al., 2002). This pattern is not limited to fMRI data, as it has been shown that the release from transcallosal inhibition between right and left SM1 (described earlier) also seems to contribute to better motor performance in older adults (Fling and Seidler, 2012; Fling et al., 2012). More recently, others have interpreted age-related increases in sensorimotor functional connectivity (FC) measures as also reflecting compensatory processes (Tomasi and Volkow, 2012; Mathys et al., 2014; Seidler et al., 2015). However, most FC analyses of the aging motor system have examined resting-state, rather than task-based data, the latter being more pertinent to the goal of this study (i.e., to examine age-related differences in the motor system as it mobilizes to perform a motor task). The second interpretation is de-differentiation (Carp et al., 2011; Bernard and Seidler, 2012), in which additional brain areas in older adults are recruited non-selectively after age-related degeneration of connections. In this case, additional activations do not correlate with performance or task demands (Riecker et al., 2006), or are possibly negatively correlated (Loibl et al., 2011). This controversy is not limited to the motor system, as it extends into vision (Park et al., 2004, 2012) and cognition (for a review, please see Grady, 2012). For example, the observation that older adults tend to show bilateral PFC activity during working memory tasks that elicit unilateral PFC activity in young adults has been attributed to compensation (Cabeza, 2002); however, to the best of our knowledge, the possibility of age-related changes in inter-hemispheric inhibition as we described in Section “Primary Sensorimotor Cortex” has not yet been investigated in the cognitive domain. This debate is also not limited to aging; interpretations of developmental changes are also controversial (Poldrack, 2010), and it is unclear whether differences in activation in children of different ages reflect compensation for immaturity or are a result of that immaturity.

As we cannot quantify performance or task demands for all studies in this meta-analysis, resolving the controversy between compensation and de-differentiation is beyond the scope of this study. Nevertheless, our observation of unique likelihoods of activations in right SM1 (extending into PMd) and SMG, three brain areas whose connections have been characterized in the animal literature (Geyer et al., 2000), suggests that right SM1 might be activated in older adults by input from SMG via PMd. This compensation by an aging-specific ipsilateral network would be the most parsimonious interpretation of our cortical results. Nevertheless, we would not exclude the possibility—as discussed earlier—that degeneration of transcallosal inhibition, as would be one basis for de-differentiation, also contributes to the unique likelihood of activation in SM1 specifically, which would indicate that both compensation and de-differentiation occur in aging.

## Conclusion

The present study quantitatively summarizes the extant literature on the brain basis of simple movements in older adults. Further, it provides comparisons between this ALE map and that of young adults. As expected, we observed likelihoods of activation in right SM1 in older adults and in the older-versus-young-adult comparison that was absent in younger adults. We also observed a unique likelihood of activation in right SMG, which, together with the concordance in right SM1 extending into PMd, suggests that older but not young adulthood is marked by recruitment of an ipsilateral network for right-hand finger movements. Importantly, we also observed a posterior shift in likelihood of activation in SMA in older age and relatively less concordance in left posterior cerebellum in older compared with young adults. This study contributes to our understanding of age-related changes in motor activity in unimpaired populations and offers a foundation for studying disease-related changes in older adults.

## Author Contributions

TT: This author contributed substantially to the conception, design, acquisition, analysis and interpretation of this work. This author drafted the majority of this work and revised other portions critically. This author approves of this version for publication and agrees to be accountable for all aspects of the work in ensuring that questions related to the accuracy or integrity of any part of the work are appropriately investigated and resolved. PT: This author contributed substantially to the design and interpretation of this work. This author revised this work critically. This author approves of this version for publication and agrees to be accountable for all aspects of the work in ensuring that questions related to the accuracy or integrity of any part of the work are appropriately investigated and resolved. GE: This author contributed substantially to the conception, design, and interpretation of this work. This author drafted portions of this work and critically revised other portions. This author approves of this version for publication and agrees to be accountable for all aspects of the work in ensuring that questions related to the accuracy or integrity of any part of the work are appropriately investigated and resolved.

## Conflict of Interest Statement

The authors declare that the research was conducted in the absence of any commercial or financial relationships that could be construed as a potential conflict of interest.

## Footnotes

1 https://www.ncbi.nlm.nih.gov/pubmed/

2 http://scholar.google.com/

3 http://www.brainmap.org

4 https://www2.warwick.ac.uk/fac/sci/statistics/staff/academic-research/nichols/software/fdr/

5 http://www.talairach.org/daemon.html

6 http://rii.uthscsa.edu/mango/
